# Monomethylsulochrin isolated from biomass extract of *Aspergillus* sp. against *Leishmania amazonensis*: *In vitro* biological evaluation and molecular docking

**DOI:** 10.3389/fcimb.2022.974910

**Published:** 2022-08-25

**Authors:** João Victor Silva-Silva, Rosiane Fernandes Moreira, Luciano Almeida Watanabe, Celeste da Silva Freitas de Souza, Daiana de Jesus Hardoim, Noemi Nosomi Taniwaki, Alvaro Luiz Bertho, Kerolain Faoro Teixeira, Arthur Ribeiro Cenci, Thiago Henrique Doring, José Wilmo da Cruz Júnior, Aldo Sena de Oliveira, Patrícia Santana Barbosa Marinho, Kátia da Silva Calabrese, Andrey Moacir do Rosario Marinho, Fernando Almeida-Souza

**Affiliations:** ^1^ Laboratory of Immunomodulation and Protozoology, Oswaldo Cruz Institute, Oswaldo Cruz Foundation, Rio de Janeiro, RJ, Brazil; ^2^ Laboratory of Medicinal and Computational Chemistry, Institute of Physics of São Carlos, University of São Paulo, São Carlos, SP, Brazil; ^3^ Post-graduate Program in Chemistry, Federal University of Pará, Belém, PA, Brazil; ^4^ Electron Microscopy Center, Adolfo Lutz Institute, São Paulo, SP, Brazil; ^5^ Flow Cytometry Core Facility, Oswaldo Cruz Institute, Fiocruz, Rio de Janeiro, RJ, Brazil; ^6^ Laboratory of Immunoparasitology, Oswaldo Cruz Institute, Oswaldo Cruz Foundation, Rio de Janeiro, RJ, Brazil; ^7^ Department of Exact Sciences and Education, Federal University of Santa Catarina, Blumenau, SC, Brazil; ^8^ Post-Graduate in Animal Sciences, State University of Maranhão, São Luís, Maranhão, Brazil

**Keywords:** monomethylsulochrin, *Leishmania amazonensis*, electron microscopy, flow cytometry, mitochondrial membrane potential, CYP51, ADMET

## Abstract

Leishmaniasis represents a serious world health problem, with 1 billion people being exposed to infection and a broad spectrum of clinical manifestations with a potentially fatal outcome. Based on the limitations observed in the treatment of leishmaniasis, such as high cost, significant adverse effects, and the potential for drug resistance, the aim of the present study was to evaluate the leishmanicidal activity of the compounds pseurotin A and monomethylsulochrin isolated from the biomass extract of *Aspergillus* sp. The chromatographic profiles of the extract were determined by high-performance liquid chromatography coupled with a diode-array UV-Vis detector (HPLC-DAD-UV), and the molecular identification of the pseurotin A and monomethylsulochrin were carried out by electrospray ionization mass spectrometry in tandem (LC-ESI-MS-MS) and nuclear magnetic resonance (NMR). Antileishmanial activity was assayed against promastigote and intracellular amastigote of *Leishmania amazonensis*. As a control, cytotoxicity assays were performed in non-infected BALB/c peritoneal macrophages. Ultrastructural alterations in parasites were evaluated by transmission electron microscopy. Changes in mitochondrial membrane potential were determined by flow cytometry. Only monomethylsulochrin inhibited the promastigote growth (IC_50_ 18.04 ± 1.11 µM), with cytotoxicity to peritoneal macrophages (CC_50_ 5.09 91.63 ± 1.28 µM). Activity against intracellular amastigote forms (IC_50_ 5.09 ± 1.06 µM) revealed an increase in antileishmanial activity when compared with promastigotes. In addition to a statistically significant reduction in the evaluated infection parameters, monomethylsulochrin altered the ultrastructure of the promastigote forms with atypical vacuoles, electron-dense corpuscles in the cytoplasm, changes at the mitochondria outer membrane and abnormal disposition around the kinetoplast. It was showed that monomethylsulochrin leads to a decrease in the mitochondrial membrane potential (25.9%, *p* = 0.0286). Molecular modeling studies revealed that monomethylsulochrin can act as inhibitor of sterol 14-alpha-demethylase (CYP51), a therapeutic target for human trypanosomiasis and leishmaniasis. Assessed for its drug likeness, monomethylsulochrin follows the Lipinski Rule of five and Ghose, Veber, Egan, and Muegge criteria. Furthermore, monomethylsulochrin can be used as a reference in the development of novel and therapeutically useful antileishmanial agents.

## Introduction

Leishmaniasis are major public health problems in developing countries. These diseases are among the endemics considered a priority in the world, being cited in 92 countries and territories, with 1 billion people being exposed to infection (WHO, 2022). In addition to the toxicity of antileishmanial drugs, another serious problem that makes the treatment of the diseases difficult is the development of resistance by the parasite (Kaye and Scott, 2011). Studies have shown isolated parasites resistant to antimonials (Sundar and Olliaro, 2007; Chakravarty and Sundar, 2010). The parasite resistance to antimonials has lead the necessity to search for new drugs. Fungi have been shown to be an excellent source for biologically active compounds with therapeutic potential, thus they could be a source of alternative treatments (Hoeksma et al., 2019).

Fungi are good producers of secondary bioactive metabolites. Some compounds with leishmanicidal activity have been isolated from fungi, such as cytochalasin B isolated from *Aspergillus* sp., which showed leishmanicidal activity against *Leishmania amazonensis* promastigotes forms (Feitosa et al., 2019). Georatusin produced by the soil fungus *Geomyces auratus* shows specific antiparasitic activities against *Leishmania donovani* and *Plasmodium falciparum* without cytotoxicity to mammalian cells (Shi et al., 2018). From the fungus *Cochliobolus* sp., it was possible to isolate anhydrocochlioquinone A (ANDC-A), which exhibited leishmanicidal activity with EC_50_ value of 22.4 µg/ml (44 µM) and antimicrobial and antitumor activity (Campos et al., 2017).

The mitochondria of *Leishmania* parasites are different from the mammalian mitochondria (Fonseca-Silva et al., 2011). Then, the mitochondrial damage can be a good target in search for new active compounds against protozoan (Fonseca-Silva et al., 2011; Pedra-Rezende et al., 2022). The antileishmanial activity of flavonoid epigallocatechin-3-gallate (EGCG) against *L. amazonensis* promastigotes is associated with mitochondrial dysfunction causing parasite death (Inacio et al., 2012). On the same way, ketoconazole (Nunes et al., 2022), carajurin (Silva-Silva et al., 2022), and dihydroartemisinin (Grazzia et al., 2021) had their antileishmanial activity associated with mitochondrial dysfunction that interfered in *Leishmania* survival.

The compound monomethylsulochrin is widely described as a metabolite of endophytic fungi from *Aspergillus* sp. (Radić and Štrukelj, 2012; Pinheiro et al., 2013; Zhang et al., 2014). This compound showed only weak antibacterial activity and no antifungal activity (Zhang et al., 2014). However, there was no report in the literature of its antiparasitic potential. Furthermore, there are few studies of compounds that isolated from the endophytic fungi from Amazon plants. In the present work, the compounds pseurotin A and monomethylsulochrin, isolated by chromatographic procedures from the biomass extract from *Aspergillus* sp., were examined against *L. amazonensis* promastigotes to pre-select active candidates. The promising compound monomethylsulochrin was subsequently tested for efficacy in intracellular amastigote assays and in tests to elucidate the mechanism of cell death induction.

## Material and methods

### Reagents

Methanol high-performance liquid chromatography (HPLC) degree (J.T. Baker, Phillipsburg, NJ, USA), Chloroform-d (deuterochloroform, CDCl3) (Cambridge, Tewksbury, MA, USA), potato dextrose agar (PDA) medium (HiMedia, Mumbai, India), ethyl acetate, hexane, and methanol were purchased from Tedia (Rio de Janeiro, Brazil). Brewer thioglycolate medium, RPMI 1640 medium, Schneider’s insect medium, amphotericin B, streptomycin, 3-(4,5-dimethylthiazol-2-yl)-2,5-diphenyltetrazolium bromide (MTT), dimethyl sulfoxide (DMSO), EPON 812 resin, glutaraldehyde, osmium tetroxide, sodium cacodylate buff;er, ferrocyanide, calcium chloride, acetone, uranyl acetate, and lead citrate were purchased from Sigma-Aldrich (St. Louis, MO, USA). Fetal bovine serum (FBS) and penicillin were acquired from Gibco (Gaithersburg, MD, USA). Tetramethylrhodamine ethyl ester (TMRE) was acquired from Molecular Probes (Carlsbad, CA, USA).

### Fungus

The strain of *Aspergillus* sp. (code FRIZ04) was obtained from a collection of the Laboratório de Bioensaios e Química de Micro-organismos (LaBQuiM), from post-graduate program in Chemistry - Federal University of Pará, and reactivated in PDA medium for work.

### Culture of *Aspergillus* sp. in rice and isolation of the compounds

Mycelia of *Aspergillus* sp. contained in small cubes of PDA medium were added in 10 Erlenmeyer flasks of 1 L, containing 200 g of rice plus 100 ml of water each, under sterile conditions. Two flasks (only with rice) were used as controls. The fungi growth in the culture medium for 28 days at 25°C and the biomass obtained were macerated with ethyl acetate. After filtration, the ethyl acetate solution was evaporated under reduced pressure producing a yellowish residue (15.2 g). A part of the ethyl acetate extract (5 g) was fractionated on chromatographic column using silica gel SiliaSphere™ (Silicycle, Québec, QC, Canada, 60–200 mesh) as stationary phase and hexane, ethyl acetate and methanol, in polarity gradient, as mobile phase, resulting in the fractions hexane/ethyl acetate 8:2 (F1), hexane/ethyl acetate 1:1 (F2), hexane/ethyl acetate 3:7 (F3), ethyl acetate (F4), and methanol (F5). To isolate the compounds 1 (65 mg) and 2 (10.3 mg), it was carried out in preparative mode from the F2 fraction using a chromatograph Waters 1525 Binary HPLC Pump (Waters, Milford, MA, USA) equipped with Waters 2998 PAD and Sunfire™ prep C18 OBD column (5 µm, 19 mm × 150 mm). Chromatographic separation occurred with 500 μl of injected volume of the sample in gradient elution H_2_O/MeOH (50–100%) for 30 min, flow of 13.0 ml/min. The scanning wavelength was in the range of 210–600 nm. Monitored wavelength was 256 nm.

### NMR and MS procedures

The mass spectra data were obtained on electrospray ionization (ESI) and atmospheric pressure chemical ionization (APCI) in positive ion mode using a Waters Acquity TQD instrument (Waters, Milford, MA, USA). One-dimensional (1D) and 2D nuclear magnetic resonance (NMR) spectra (Bruker, Fällanden, Switzerland) were recorded on a Bruker Ascend 400, using chloroform-d3 solvent to dilution of the compounds. The chemical shifts are given in delta (δ) values and the coupling constants (J) in Hertz (Hz).

### Parasites

Promastigote forms of *L. amazonensis* H21 (MHOM/BR/76/MA-76) were cultured at 26°C in Schneider’s insect medium. The culture was supplemented with 10% FBS, 100 IU/ml of penicillin, and 100 µg/ml of streptomycin in a maximum of 10 *in vitro* passages (Almeida-Souza et al., 2016).

### Animals

The experimental procedures used female BALB/c mice aged 4–6 weeks, acquired from the Institute of Science and Technology in Biomodels from Oswaldo Cruz Foundation.

### Peritoneal macrophage collection and culture

The animals were stimulated by intraperitoneal inoculation of 3.0 ml of sodium thioglycolate at 3.0%. After 72 h of elicitation, the animals were euthanized with 10% ketamine (Syntec, São Paulo, BRA) and 2% xylazine (Syntec, São Paulo, BRA) according to the weight of each animal and, after death, the animal’s skin was retracted by incision and subsequent divulsion for peritoneum exposure. A total of 10.0 ml of sterile pH 7.2 phosphate-buffered saline (PBS) was inoculated, and a light manual massage was performed. The cells were then harvested from the peritoneum and dispensed in a sterile conical tube. The peritoneal lavage was centrifuged at 380*g* for 5 min, and cells were suspended in culture medium (RPMI 1640 medium supplemented with 10% FBS, penicillin [100 U/ml], and [100 μg/ml] streptomycin) and incubated at 37°C, 5% CO_2_ overnight (Almeida-Souza et al., 2018).

### Antileishmanial activity assay

In 96-well plates, the promastigote forms of *L. amazonensis* (10^6^ parasites/ml) from a 3- to 5-day-old culture were incubated for 24 h in the presence of different concentrations of pseurotin A at 7.24–579.5 μM and monomethylsulochrin at 9.0–721.9 μM, in a final volume of 100 µL per well. The viability of parasites was evaluated after treatment by counting the total number of live promastigotes, considering the flagellar motility, using Neubauer’s camera and optical light microscope (Rottini et al., 2019). This count was compared with the score of non-treated promastigote growth. This experiment was carried out in independent triplicate with each condition performed in triplicate. The results were expressed as parasite growth inhibitory concentration of 50% (IC_50_). Wells with medium and without parasites were used as blanks, and wells with parasites only incubated with medium were used as controls. Amphotericin B at 0.0334–1.0 μM was used as reference drug. Activity against intracellular amastigote was performed in 24-well plates, with coverslips, with peritoneal macrophages cultured (5 × 10^5^ cells per well) and infected with promastigote forms of *L. amazonensis* using a ratio of 10:1 parasites per cell. After 6 h, the cells were washed three times with PBS to remove free parasites. The infected cells were treated with monomethylsulochrin at 0.361–5.78 μM, or amphotericin B at 0.0845–2.7 μM for 24 h. For the light microscopy analysis, the coverslips with the infected and treated cells were then fixed with Bouin solution and stained with Giemsa. The IC_50_ values of all compounds tested were calculated from the total of intracellular amastigote from 100 cells. Parameters of infection analysis were performed according to Silva-Silva et al. (2021a).

### Cytotoxicity assay and selectivity index

Peritoneal macrophages were cultured in 96-well plates (5 × 10^5^ cells/ml) with different concentrations of pseurotin A at 9.0–1159 μM, monomethylsulochrin at 5.63–721.9 μM, or amphotericin B at 0.211–27 μM in a final volume of 100 μl per well, at 37° C and 5% CO_2_. Wells without cells were used as blank, and wells with cells and 1% DMSO were used as control. After 24-h incubation period, cell viability was assessed by MTT. The cytotoxicity of 50% (CC_50_) was calculated following the method described elsewhere by Oliveira et al. (2018). The selectivity index (SI) was obtained from the ratio of peritoneal macrophages CC_50_ and IC_50_.

### Transmission electron microscopy

The transmission electron microscopy was performed to evaluate if monomethylsulochrin induced any ultrastructural alterations to the *L. amazonensis* promastigote forms. Parasites were treated with monomethylsulochrin with IC_50_ for 24 h and fixed with 2.5% glutaraldehyde in sodium cacodylate buff;er (0.1 M, pH 7.2) for overnight, then washed three times with 0.1 M sodium cacodylate buffer and postfixed in a solution containing 1% osmium tetroxide, 0.8% ferrocyanide, and 5 mM calcium chloride. 0.1 M sodium cacodylate buffer was used for washing, before being dehydrated in graded acetone and embedded in EPON 812 resin. Ultrathin sections were stained with 5% uranyl acetate aqueous solution and lead citrate (1.33% lead nitrate and 1.76% sodium citrate) and examined in a JEM-1011 transmission electron microscope (JEOL, Tokyo, Japan) at 80 kV (Silva-Silva et al., 2021b). The images acquired were used for quantification of the number of vacuoles and lipid corpuscles and area of vacuoles, lipid corpuscles, and mitochondria, using ImageJ software (NIH). Quantitative analysis was conducted with five untreated parasites and at least 10 treated parasites. The number of vacuoles and lipid corpuscles was expressed as mean ± standard deviation (*SD*), and the area of vacuoles, lipid corpuscles, and mitochondria were expressed as percentage of each parasite total area.

### Flow cytometry

The promastigote forms of *L. amazonensis* (2 × 10^6^ cells/ml) were treated with monomethylsulochrin IC_50_ for 24 h at 26°C. Heat-killed parasites (60°C bath for 60 min) and untreated parasites were used as positive and negative controls, respectively. After incubation, parasites were centrifuged at 210*g* for 5 min at room temperature, washed in PBS, incubated with 300 μl of TMRE (50 nM) in the dark for 15 min, at room temperature, and submitted to flow cytometry through CytoFlex flow cytometer (Beckman Coulter Life Sciences, IN, USA). TMRE was excited by 488-nm blue laser, and its fluorescence was collected at 585/42 nm bandpass filter. CytExpert software version 2.1 (Beckman Coulter Life Sciences, IN, USA) was used for flow cytometry analyses (Mondêgo-Oliveira et al., 2021).

### Molecular docking

#### Homology

The monomethylsulochrine molecule was downloaded from the ChemSpyder database and optimized *via* Avogadro (version 1.2.0) with UFF (Universal Force Field) up to dE = 1 × 10–12 kJ/mol and exported as.mol2 (Hanwell et al., 2012). Docking by homology was performed using the GOLD program Hermes 2021.3.0 (Jones et al., 1997), changing the structure of CYP51 (*L. infantum*) *via* SWISS-MODEL (Waterhouse et al., 2018) to *L. amazonensis* using the highest *GMQE* gene, which was the LAMA_000181200 from Cytochrome P450 (putative), available from TriTrypDB.org (Amos et al., 2022). The structure chosen was a 3TIK - Sterol 14-alpha demethylase (CYP51) from *Trypanosoma brucei* in complex with the derivative tipifarnib 6-([4-chlorophenyl] [methoxy][1-methyl-1H-imidazol-5-yl]methyl) -4-(2,6-difluorophenyl)-1-methylquinolin-2(1H) (Buckner et al., 2012), which showed *GMQE* of 0.85, identity of 79.19% and resolution of 2.05 Angstroms.

Hydrogens were added, as water and co-crystallized ligands removed and the binding site was assembled using the HEM3 ligand as a starting point, on a 10 Å sphere. The rest of the settings were kept as default. The redocking overlap was performed for 3TIK, with a root-mean-square deviation (RMSD) close to 1 Å in relation to the co-crystallized ligand, with the ligands showing better behavior in the GoldScore evaluation. Docking by homology with an altered protein was performed under the same conditions, with RMSD shift also predominantly less than 1.

#### Protein-ligand interaction

To perform the molecular docking simulation, the structure of the murine inosoxy dimer from the protein data bank (PDB 1DF1, 2.35 Å) was considered rigid and the binders were treated as fully flexible. The optimization of the ligands and the assignment of their atomic charges were performed by the program VEGA ZZ v.3.2 (Hanwell et al., 2012). In the ligand optimization process, a steeper descent algorithm was used (100 steps. FF: AM1BCC) and the Ammp-Mom method was used to assign the charges. The receptor preparation was performed using the GOLD v.2021.3.0 program, requiring the use of only the A chain of the dimer. Crystallographic water molecules were not considered.

The calculation of the enzyme-substrate interaction was also performed by the program GOLD v.2021.3.0 (Jones et al., 1997). The definition of the binding site was based on the position of the reference crystallographic inhibitor (H4B), considering residues that were at up to 6 Å from the center of H4B. The scoring functions used were ChemPLP, ChemScore, ASP, and GoldScore. The GOLD program was configured to present the 10 best poses for each ligand. The choice of the results of the different scoring functions used took into account the best scores of the most populous pose groupings. The standard parameters for cavity detection and PDB data were used, allowing R-H rotations and [O, N, S]-H bonds. Receptor-ligand figures were generated with PyMOL v.1.8.

### *In silico* pharmacokinetics prediction

The 2D structure of the compounds was obtained by ChemDraw software (version Ultra 12.0, PerkinElmer Informatics, Waltham, MA, USA) and were converted into a single database file SMILES. *In silico* physicochemical and pharmacokinetics predictions were made using two different tools: pkCSM (Pires et al., 2015) and SwissADME (Daina et al., 2017).

### Statistical analysis

The values were expressed as mean ± *SD*. The IC_50_ and CC_50_ were determined using GraphPad Prism^®^ version 7 software (GraphPad Software Inc., San Diego, CA, USA), and the differences were considered significant when *p* < 0.05 by one-way analysis of variance (ANOVA) or Mann–Whitney test.

## Results and discussion

### Isolation of the compounds

**Figure 1:**
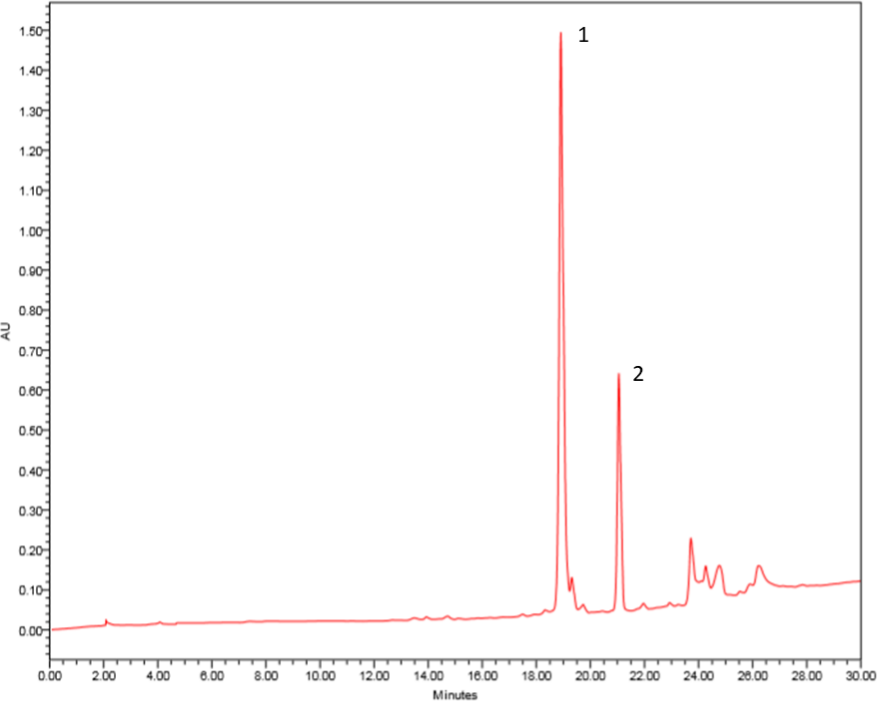
Chromatogram of F2 fraction of ethyl acetate extract obtained from *Aspergillus* sp. biomass. HPLC-DAD in preparative mode using a chromatograph Waters 1525 Binary HPLC Pump equipped with Waters 2998 DAD and Sunfire™ prep C18 OBD column (5 µm, 19 mm × 150 mm), 500 μl of injected volume, gradient elution H_2_O/MeOH (50–100%) for 30 min, flow of 13.0 ml/min. The wavelength scanning was in the range of 210–600 nm. Pseurotin A (1) and monomethylsulochrin (2).

The compounds 1 and 2 were isolated from F2 fraction of ethyl acetate extract obtained from *Aspergillus* sp. biomass by preparative HPLC-DAD. The compound 1 was observed at 19 min, and the compound 2 was isolated at 21 min (**Figure 1**).

### Chemical constituent identification

**Figure 2:**
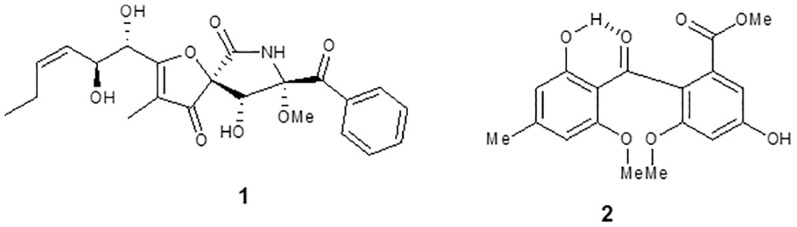
Chemical structure of compounds isolated from *Aspergillus* sp. (1) pseurotin A and (2) monomethylsulochrin.

The compound 1 (**Figure 2**) was isolated as a crystalline solid soluble in dichloromethane. The mass spectrum ESI (+) showed a m/z 432 [M+H]+, which combined with NMR data allowed to propose the molecular formula C_22_H_25_O_8_N. The ^1^H NMR spectrum showed signals typical to aromatic ring monosubstituted at δ 8.31 (7.8 Hz, H-19/23), δ 7.49 (7.5 Hz, H-20/22), and δ 7.65 (7.2 Hz, H-21). A spiro bicycle system was confirmed through of heteronuclear multiple bond correlation (HMBC) of signal at δ 4.69 (s, H-9) with δ 196.6 (C-4), 92.7 (C-5), 166.8 (C-6), 90.6 (C-8), and 194.9 (C-17). Signals to unsaturated side chain dihydroxylated at H-10 δ 4.58 (d, *J* = 5.6 Hz) and H-11 δ 4.75 (dd, *J* = 8.8; 0.9) were observed. Moreover, signal to methoxyl group was observed at δ 3.44 (s). Following, HMBC correlation allowed close the lactam ring, and the acetophenone group was fixed at C-8. Compound 1 was identified as the alkaloid pseurotin A (Maiya et al., 2007).

The compound 2 (**Figure 2**) was isolated as a pale yellow crystalline solid. The APCI (+) mass spectrum of compound 2 showed m/z 347 [M+H]+, which along with NMR data has led to the molecular formula C_18_H_18_O_7_. The ^1^H NMR spectrum showed signals compatible for two aromatic rings. In ^13^C, NMR spectrum still observed a signal at δ 199.4 assigned to benzophenonic carbonyl group. Through the HMBC correlations of OMe-9 and H-6 with the signal at 166.1, the acetate group was positioned at C-1. HMBC correlations of OH-2’ and H-3’ with the signal at 164.1 allowed us to locate the hydroxyl group at C-2’. Furthermore, the correlations of Me-7’ with the C-3’ and C-5’ carbons allowed closing the 2-hydroxy-4-methyl-acetophenone moiety in the molecule. Thus, the compound 2 was identified as benzophenone monomethylsulochrin (Ma et al., 2004).

### Antileishmanial activity and cytotoxicity

Pseurotin A (**1**) and monomethylsulochrin (**2**) were tested for their cytotoxicity against mammal cells, as well as their inhibitory effect against the promastigote and intracellular amastigote forms of *L. amazonensis* (**Table 1**). Analyzing the activity against different forms of the parasite, only monomethylsulochrin (**2**) showed an inhibitory capacity, with an IC_50_ value against intracellular amastigotes 3.5 times higher compared with the IC_50_ against promastigote forms. When observing cytotoxicity, it can be seen that monomethylsulochrin showed greater selectivity against intracellular forms in relation to the reference drug amphotericin B (**Table 1**).

**Table 1:** Antileishmanial activity, cytotoxicity in BALB/c peritoneal macrophages, and selectivity index after 24 h of treatment with compounds from *Aspergillus* sp. and amphotericin B.

Compounds	Cytotoxicity	*L. amazonensis*			
	CC_50_ (µM)	Promastigote		Intracellular amastigote	
		IC_50_ (µM)	SI_pro_	IC_50_ (µM)	SI_ama_
Pseurotin A	654.38 ± 1.19	> 500	ND	ND	ND
Monomethylsulochrin	91.63 ± 1.28	18.04 ± 1.11	5.08	5.09 ± 1.06	18.00
Amphotericin B	8.45 ± 1.08	0.027 ± 1.14	312.96	0.629 ± 1.39	13.43

Data represents mean ± SD of at least two experiments done in triplicate; CC_50_: cytotoxic concentration for 50% of peritoneal macrophage; IC_50_: inhibitory concentration for 50% of parasites; SI_pro_ (selectivity index) = CC_50_ macrophages/IC_50_ promastigote forms. SI_ama_ (selectivity index) = CC_50_ macrophages/IC_50_ amastigote forms; ND: not determined.

**Figure 3:**
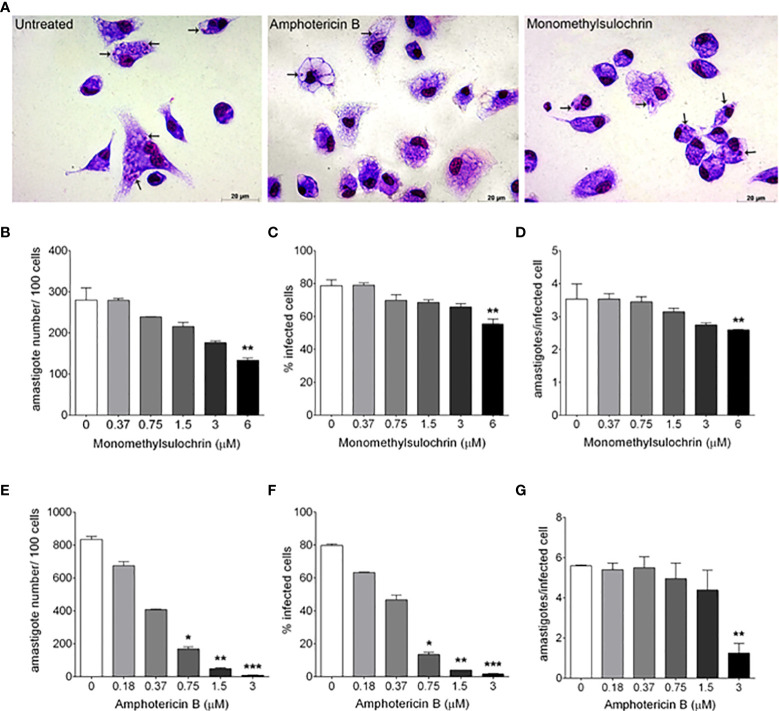
*In vitro* effects of monomethylsulochrin and amphotericin B against *Leishmania amazonensis* after 24 h of treatment. Light microscopy 24 h after amphotericin B or monomethylsulochrin treatment at 0.75 or 6 μM, respectively **(A)**. Infection parameters of BALB/c peritoneal macrophages infected with *L. amazonensis* and treated with monomethylsulochrin **(B–D)** or amphotericin B **(E–G)**. Intracellular amastigotes inside macrophages (arrows). Data represent mean ± *SD* of two independent experiments realized in triplicate. *p < 0.05, **p < 0.01, and ***p < 0.001 when compared with the untreated group by the Mann–Whitney test.

**Figure 3A** shows the photomicrographic images of the decrease in the number intracellular amastigotes of *L. amazonensis* after treatment with monomethylsulochrin and amphotericin B. The analysis of parameters of infection showed that monomethylsulochrin at 6 µM induced a statistically significant reduction of parameters of infection, with decrease of amastigote number in 100 cells (*p* = 0.0082; **Figure 3B**), percentage of infected cells (*p* = 0.0033; **Figure 3C**), and the mean of amastigotes per infected cell (*p* = 0.0041; **Figure 3D**). Amphotericin B showed a significant reduction in all infection parameters at 3 µM (**Figures 3E–G**).

Promastigote forms were used in conjunction with cytotoxicity analysis to screen active compounds. Only monomethylsulochrin showed activity against *L. amazonensis* promastigote. Martińez-Luis et al. (2012) previously reported the activity of the compound pseurotin A, isolated from endophytic *Aspergillus* sp. strain F1544, against axenically cultured (cell free) *L. donovani* amastigotes (IC_50_ 5.8 µg/ml). Almeida-Souza et al. (2018), using the same methodology, observed that the IC_50_ of *Morinda citrifolia* fruit juice for the axenic amastigote forms was lower than for the *L. amazonensis* promastigotes. The genomic microarray of promastigotes and axenic amastigotes shows variation in gene expression that influence stress response and metabolism (Saxena et al., 2007; Srividya et al., 2007; Dias-Lopes et al., 2021). Therefore, the difference obtained in the screening of compounds evaluated against *Leishmania* could be due to the difference between the life cycle stages of the parasite. This may help explain the differences between our results and those described by Martińez-Luis et al. (2012).

Literature data report the activity of the benzophenone class against different species of *Leishmania*. Studies by Hay et al. (2008) showed that the compound 3-geranyl-2,4,6-trihydroxybenzophenone presented a significant antileishmanial activity against the promastigote forms of *L. mexicana* (IC_50_ 4.05 μg/ml) and *L. infantum* (IC_50_ 15 μg/ml), as well as against the intracellular amastigote forms of *L. infantum* (IC_50_ 0.7 μg/ml). Other studies performed with a series of synthetic benzophenones, structurally similar to monomethylsulochrin, tested the activities of these compounds against promastigote and amastigote forms of *L. amazonensis*. They found that the derivatives presented better activities than their hydroxylated precursors, which led to infer that the decrease in the polarity of the derivatives is one of the parameters associated with the antileishmanial activity improvement observed (Maciel-Rezende et al., 2013; de Almeida et al., 2015). Our results in which monomethylsulochrin, a benzophenone of intermediate polarity, was active against promastigote forms (18.04 µM) and three and a half times (5.09 µM) more active against intracellular forms of *L amazonensis* support the data demonstrated by Maciel-Rezende et al. (2013) and de Almeida et al. (2015). Therefore, in theory, improving the lipophilicity could facilitate protozoa membrane permeation of these substances.

### Ultrastructural changes

**Figure 4:**
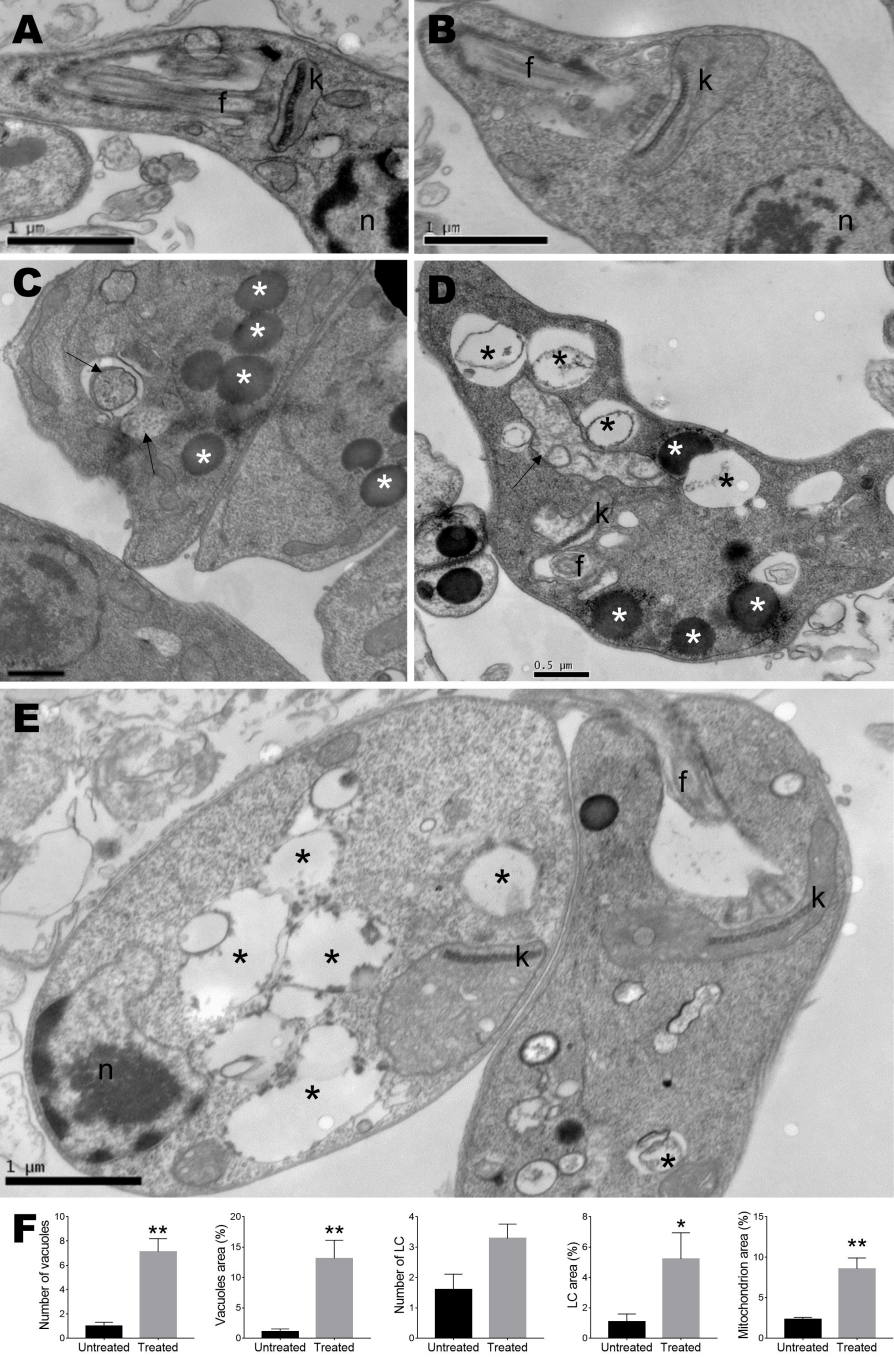
Ultrastructural analysis of *Leishmania amazonensis* promastigote forms. **(A)** Untreated parasites. **(B–E)** Parasites treated with monomethylsulochrin (18.04 μM) for 24 h. **(F)** Statistical differences data in the quantification of the vacuoles, lipid corpuscles, and mitochondria damage between the cells in the untreated group and the groups treated with monomethylsulochrin are shown. Images show several vacuoles dispersed in the cytoplasm (black asterisks), lipid corpuscles (white asterisks), vesicles with electron-dense material inside (arrows), and changes at the mitochondria outer membrane and abnormal disposition around the kinetoplast. f: flagellum; k: kinetoplast; n: nucleus; LC: lipid corpuscles. Images are representative of two independent experiments from five untreated and at least 10 treated cells. Data are presented as mean ± *SD*, and the difference between treated and untreated group was evaluated by Mann–Whitney test with *p > 0.05 and **p > 0.01.

To determine the ultrastructural changes, transmission electron microscopy analysis of *L. amazonensis* promastigotes treated with monomethylsulochrin was performed. Photomicrographs of promastigotes showed untreated parasites with normal morphology (**Figure 4A**) and parasite damage after 24 h of treatment with monomethylsulochrin at the IC_50_ concentration (18.04 μM). Atypical vacuoles (**Figures 4D, E**) and some electron-dense corpuscles were observed in the cytoplasm of the promastigote forms (**Figures 4C, D**), as well as changes at the mitochondria outer membrane and abnormal disposition around the kinetoplast (**Figures 4B, E**). As shown in **Figure 4F**, statistically significant changes in number of vacuoles (*p* = 0.0016), vacuoles area (*p* = 0.0016), lipid corpuscles area (*p* = 0.0480), and mitochondria area (p = 0.0079) were observed after treatment with monomethylsulochrin.

Ultrastructural changes in *L. amazonensis* promastigotes treated with the monomethylsulochrin were revealed by transmission electron microscopy. The treatment showed the presence of intense cytoplasmic vacuolization. Related studies described by Britta et al. (2014) report that 4-nitrobenzaldehyde thiosemicarbazone (BZTS), derived from S-limonene, altered the ultrastructure of the promastigote and axenic amastigote forms of *L. amazonensis*. Changes induced by the drug include damage to mitochondria, reflected by extensive swelling and disorganization of the inner mitochondrial membrane, intense cytoplasmic vacuolization, and the presence of concentric membrane structures inside the organelle (Britta et al., 2014). Herein, another important effect of monomethylsulochrin on parasites was the accumulation of intracellular lipid bodies in the cytoplasm. The presence of intracellular lipid bodies may indicate changes in phospholipids and sterol content (Medina et al., 2012; Godinho et al., 2013). Changes in mitochondria, such as outer membrane presenting swelling and abnormal disposition around the kinetoplast, were also observed. These mitochondrial changes were also seen in *Trypanosoma cruzi* epimastigotes (Y strain) treated with the of two μ-oxo Fe(III) dinuclear complexes (Moreira et al., 2021) and in *L. donovani* promastigotes treated with benzophenone-derived bisphosphonium salts, accompanied by a decrease in electrochemical mitochondrial potential (Luque-Ortega et al., 2010). In addition, changes in the ultrastructure indicated the presence of several vesicles. Thus, the association between multivesicular bodies and mitochondrial profiles probably indicates an autophagic process that removes damaged organelles (Britta et al., 2014).

### Mitochondrial membrane potential (Δψm)

**Figure 5:**
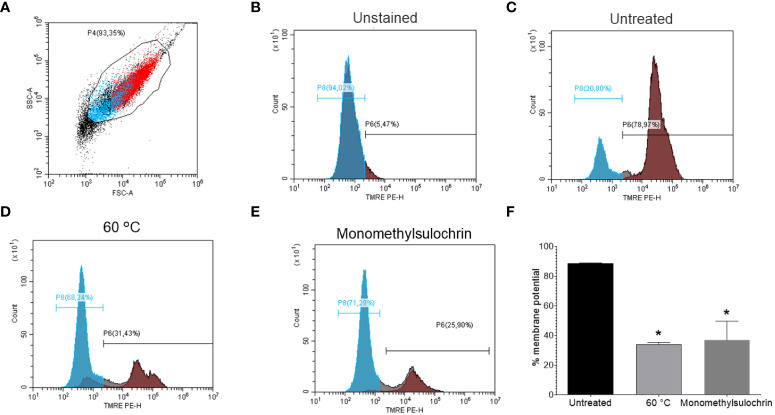
Flow cytometry of promastigote forms of *Leishmania amazonensis* mitochondrial membrane potential (ΔΨm) treated with monomethylsulochrin. **(A)** FSC versus SSC dot plot to define *L. amazonensis*-promastigotes population. **(B)** TMRE-staining histogram of control samples (unstained and untreated parasites), gated on “promastigotes.” **(C)** TMRE-staining histogram of monomethylsulochrin-untreated parasites. **(D)** *L. amazonensis* promastigotes killed by heat (60°C). **(E)** Representative histogram of *L. amazonensis* promastigotes treated with 18.04 μM of monomethylsulochrin. **(F)** Statistically significant differences were observed between the percentages of cells marked with TMRE in the untreated group and the groups treated with monomethylsulochrin, at the IC_50_ concentration (18.04 μM). *p < 0.05, when compared with the untreated group by Mann–Whitney test. Images are representative of two independent experiments carried out at least in triplicate.

Maintaining the proper potential of the mitochondrial membrane is vital to the process of energy production by cells (Joshi and Bakowska, 2011). Based on this, a flow cytometric analysis was performed in order to monitor parasite mitochondrial damage when submitted to treatment for 24 h with 18.04 μM monomethylsulochrin. We used TMRE, a cell-permeant fluorescent dye that is readily sequestered by active mitochondria (Crowley et al., 2016). **Figure 5** shows promastigotes captured in the gated region and representative histogram (**Figure 5A**) and unstained parasites (**Figure 5B**). Monomethylsulochrin*-*untreated-*L. amazonensis* promastigotes showed 78.97% of cells stained with TMRE (**Figure 5C**). When we treated parasites with heat (60°C) and 18.04 μM of monomethylsulochrin, it was observed a significant decrease in the Δψm in *L. amazonensis* promastigotes (**Figure 5D, E**). Statistically significant changes in mitochondrial membrane potential were observed after treatment with monomethylsulochrin IC_50_ (*p* = 0.0286; **Figure 5F**).

*Leishmania* has a single mitochondria, thus mitochondrial dysfunction and energy metabolic plasticity in *Leishmania* is more restricted when compared with thousands of mitochondria that can be detected in mammalian cells (Menna-Barreto and de Castro, 2014; Dagnino et al., 2018). In this regard, when considering cytotoxicity against macrophages, monomethylsulochrin showed selectivity index of 18.0. Literature data refer that the effectiveness of a compound is indicated by the selectivity index ≧̸10 (Nava-Zuazo et al., 2010). In our study, treatment with monomethylsulochrin reduced the viability of promastigotes and one of the alterations observed was the induction of mitochondrial dysfunction. It is known that impairment of the membrane potential allows compounds to cross the mitochondrial membrane, which can lead to the death of the parasite (Rottini et al., 2015). Synthetic benzophenones containing more lipophilic radicals are studied in order to increase their antileishmanial activity (Maciel-Rezende et al., 2013). Thus, highly lipophilic compounds, with the ability to cross cell and parasite membranes to reach their target, are essential for their leishmanicidal activity, since *Leishmania* spp. are a mandatory intracellular parasite (Rottini et al., 2015). Therefore, this ability can assist in understanding the activity against intracellular amastigote forms when treated with monomethylsulochrin.

Taken together, our results demonstrated that the benzophenone monomethylsulochrin presented activity against the promastigote and intracellular amastigote forms. To the best of our knowledge, this is the first time that the biomass of *Aspergillus* has been shown to have activity against *L. amazonensis.* In addition, remarkable alterations on the ultrastructure of this parasite were observed, with mitochondrial dysfunction and accumulation of bodies, characteristics found in cells that die from autophagic processes (Britta et al., 2014).

### Molecular docking

The cited advances in the areas of genomics and proteomics allowed the identification of metabolic pathways and important enzymes of *Leishmania* and led to the knowledge of their differences in relation to human metabolic pathways. Among them, the enzyme 14α-sterol demethylase (CYP51) (E.C. 1.14.13.70) stands out, which is a member of the cytochrome P450 (CYP) superfamily and transforms lanosterol into ergosterol in kinetoplastids such as *Leishmania* spp (Lepesheva and Waterman, 2011). In this work, we investigate a possible interaction between monomethylsulochrin and CYP51, a validated target for the design of new leishmanicidal agents (McCall et al., 2015) and tried to elucidate a possible mechanism of leishmanicidal action using of molecular docking. The calculations of the enzyme-substrate interaction, between the monomethylsulochrin and the CYP51 structure (PDB ID 3TIK), were performed considering the different scoring functions and the one that presented the best results was GoldScore. For this scoring function, it was possible to observe that the average value of the scores of the 10 calculated poses was approximately 48.85 ± 3.87 for the original CYP25, whereas for the edited, the average value was around 46.85 ± 0.97. The resulting RMSD shift for homology docking was predominantly less than 1.0 Å.

**Figure 6:**
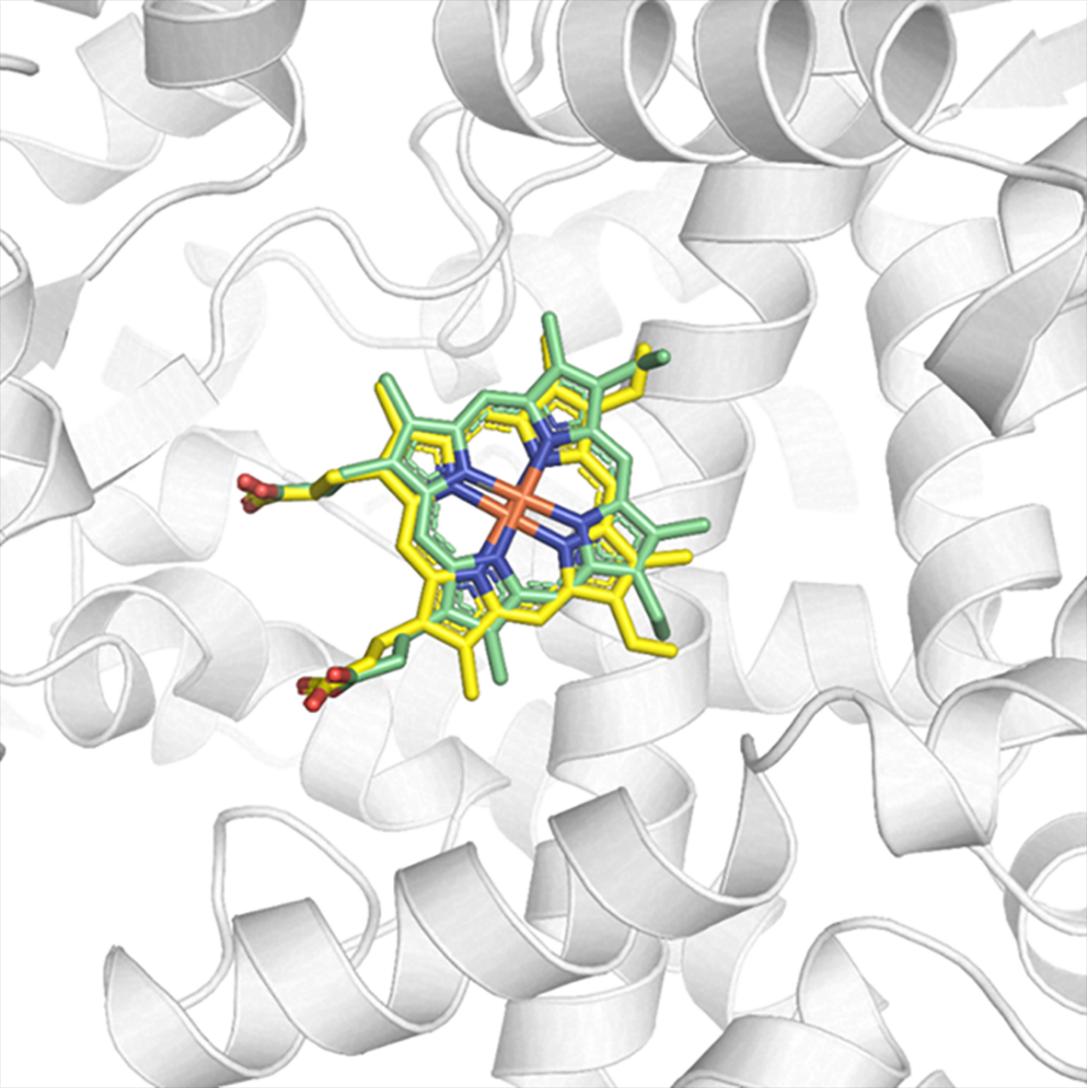
Redocking of the co-crystallized ligand HEM on the active site of CYP51 (PDB ID 3TIK). In green, the best pose of the co-crystallized ligand generated by the ChemPLP function and, in yellow, its crystallographic conformation.

Redocking studies were performed to characterize the coherence of the docking calculations performed (**Figure 6**). To verify the best redocking pose, the score values of the series with the best alignment between the generated poses were analyzed. As can be seen in **Figure 6**, the overlap between the best pose of the co-crystallized ligand—from the docking calculation—and its crystallographic position is excellent. In this sense, there are subsidies that explain the success of the applied calculation, as well as the choice of the best operational conditions for the development of this methodology.

**Figure 7:**
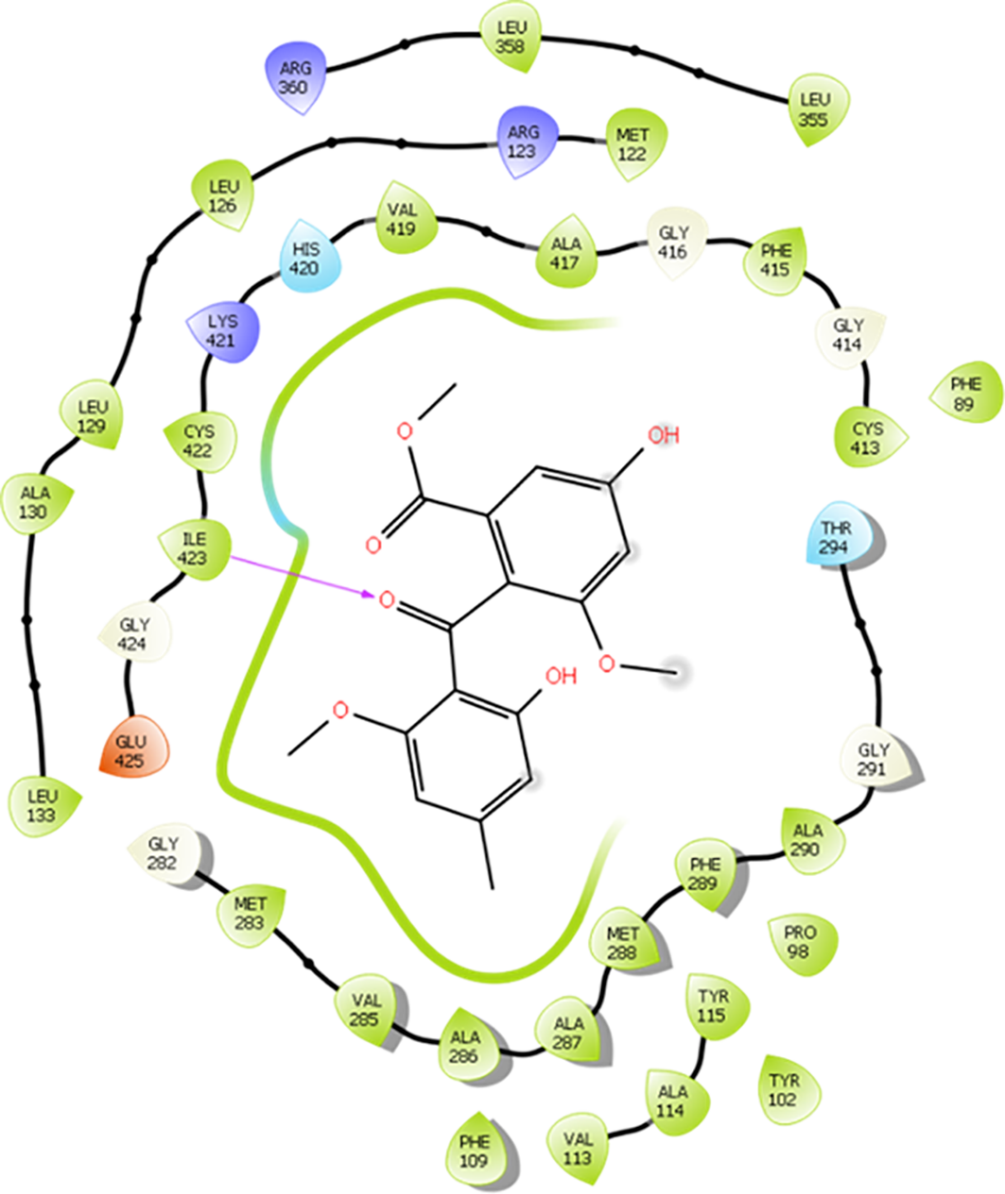
2D diagram of the interaction of monomethylsulochrine at the active site of CYP51 (PDB ID 3TIK). Hydrogen bonds are shown as arrows in purple, hydrophobic interactions in green lines, and polar interactions in light blue lines.

**Figure 8:**
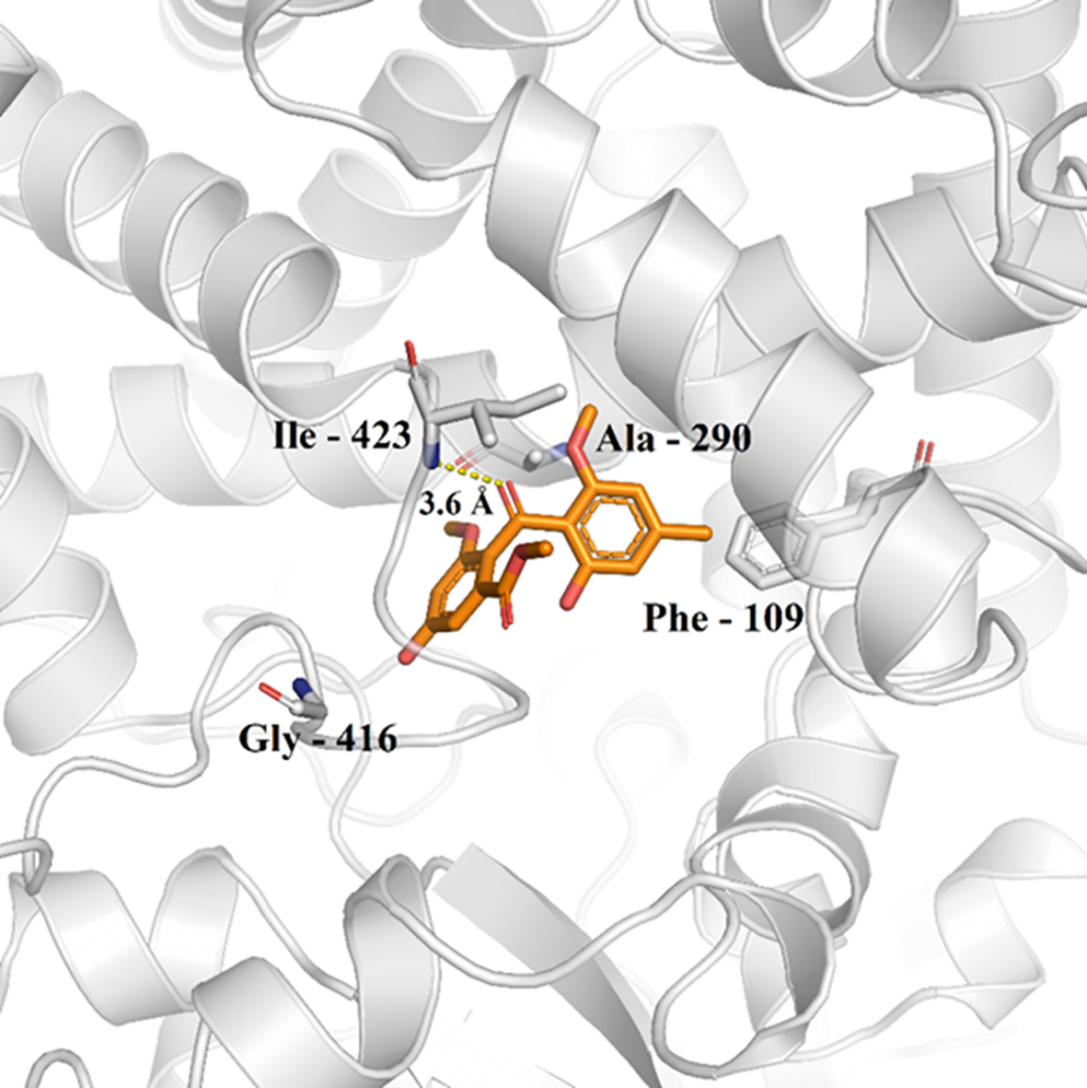
Monomethylsulochrine (carbon represented as orange sticks) in the active site of CYP51 (PDB ID 3TIK). Hydrogen bonds are shown as dashed lines.

Possible chemical interactions that justify the interaction of monomethylsulochrin with the active site of the 3TIK protein can be seen in **Figures 7**, **8**. According to the 2D diagram generated by the Maestro program (**Figure 7**), it is notable that, in addition to the possibility of an interaction of the hydrogen bond type with residue Ile–423, monomethylsulochrin would also be able to establish a long chain of hydrophobic interactions with the active site of the protein. Studies reported in the literature, with the structure of CYP51, revealed that the hydrophobic interactions that its catalytic crack is capable of performing can be considered the main driving force for the binding of ligands (Dong et al., 2019; Shi et al., 2020).

Analyzing the 3D interaction diagram (**Figure 8**), it is possible to observe that the structure of monomethylsulochrin is easily allocated to the active site of CYP51, favoring the enzyme-substrate interaction to be less sensitive to the solvent of the biological medium (Dong et al., 2019). This fact suggests that the parameters of the docking calculation performed here were correctly selected, aiming at good results and a lower computational cost.

### *In silico* drug likeness, pharmacokinetics, and toxicity prediction

*In silico* predictions of drug similarity were made with monomethylsulochrin, which showed the best Antileishmanial activity. **Table 2** describes the physico-chemical, drug likeness, and medicinal chemical properties for monomethylsulochrin and amphotericin B, a reference drug available against leishmaniasis. The physical–chemical characteristics of the topological polar surface area (TPSA) and lipophilicity of monomethylsulochrin were inferior to amphotericin B. The drug probability rules, such as the Lipinski rule of five and the rules of Veber Ghose, Muegge, and Egan, were applied to the compounds. The results obtained showed that monomethylsulochrin obeyed all the rules. In the prediction of medicinal chemistry properties, two complementary pattern recognition methods were used that allow the identification of potentially problematic fragments: pan test interference compounds (PAINS) and Brenk filters. No alerts created by the PAINS assessment and Brenk’s analysis. The lead similarity criteria, which predict whether a molecular entity is suitable for optimization, identified the monomethylsulochrin compound as a good lead compound. On the other hand, the low value of synthetic accessibility indicates that monomethylsulochrin can be easily synthesized. These results suggest that the monomethylsulochrin compounds have drug-like properties.

**Table 2:** Predicted physicochemical, drug-likeness, and medicinal chemistry properties for monomethylsulochrin compound and amphotericin B.

Property/Model Name	Compounds	
	Monomethylsulochrin	Amphotericin B
**Physicochemical**		
Molecular Weight	346.335	924.091
# Rotatable bonds	6	3
# H-bond acceptors	7	18
# H-bond donors	2	12
Surface Area	143.930	380.536
TPSA (Å2)	102.29	319.61
Lipophilicity (Log Po/w)	2.71	3.76
**Drug-likeness**		
Lipinski	Yes; 0 violation	No; 3 violations: MW>500, NorO>10, NHorOH>5
Ghose	Yes	No; 3 violations: MW>480, MR>130, #atoms>70
Veber	Yes	No; 1 violation: TPSA>140
Egan	Yes	No; 1 violation: TPSA>131.6
Muegge	Yes	No; 4 violations: MW>600, TPSA>150, H-acc>10, H-don>5
**Medicinal chemistry**		
PAINS	0 alert	0 alert
Brenk	0 alert	0 alert
Lead-likeness	Yes	No; 1 violation: MW>350
Synthetic accessibility	2.98	10.00

#, number; TPSA, topological polar surface area; PAINS, pan-assay interference compounds; MW, molecular weight.

In a complementary way, pharmacokinetic properties and toxicity parameters were predicted for the compound monomethylsulochrin and for amphotericin B (**Table 3**). The compound is predicted to have moderate water solubility, high Caco-2 permeability, high intestinal absorption, low skin permeability, and P-glycoprotein I inhibition and predicted as P-glycoprotein substrates, but it was not predicted as P-glycoprotein II inhibitior. Human steady state volume of distribution (ssVD) was low, just as it was not predicted to be readily distributed to the brain through the blood–brain barrier (BBB) and penetrate the central nervous system (CNS). Monomethylsulochrin did not show metabolization in any of the evaluated isoforms. Likewise, it was not envisaged as a renal organic cation transporter 2 (OCT2) substrate but presented total clearance. In toxicity parameters prediction, monomethylsulochrin showed high human maximum tolerated dose (>0.477 log mg/kg per day). It was also observed adverse effect (LOAEL) in high-oral acute and chronic toxicity in rat, equally it exhibited toxicity to flathead minnows and *Tetrahymena piriformis*, but no mutagenic or carcinogenic potential action by *Salmonella*/microsome mutagenicity assay (AMES) prediction, or human ether-a-go-go (hERG I and II) inhibition, or hepatotoxicity or skin sensitization was observed.

**Table 3:** *In silico* pharmacokinetics and toxicity properties of monomethylsulochrin compound and amphotericin B.

Property	Model Name	Compounds	
		Monomethylsulochrin	Amphotericin B
Absorption	Water solubility (log mol/L)	-4.129	-2.937
	Caco-2 permeability (log Papp in 10^−6^ cm/s)	1.136	-0.597
	Intestinal absorption – human (% Absorbed)	76.803	0
	Skin Permeability (log Kp)	-2.761	-2.735
	P-glycoprotein substrate	Yes	Yes
	P-glycoprotein I inhibitor	Yes	No
	P-glycoprotein II inhibitor	No	No
Distribution	Human ssVD (log L/kg)	-0.344	-0.37
	BBB permeability (log BB)	-0.416	-2.058
	CNS permeability (log PS)	-3.138	-3.718
Metabolism	CYP2D6 substrate	No	No
	CYP3A4 substrate	No	No
	CYP1A2 inhibitor	No	No
	CYP2C19 inhibitor	No	No
	CYP2C9 inhibitor	No	No
	CYP2D6 inhibitor	No	No
	CYP3A4 inhibitor	No	No
Excretion	Total Clearance (log ml/min/kg)	0.724	-1.495
	Renal OCT2 substrate	No	No
Toxicity	AMES toxicity	No	No
	Human max. tolerated dose (log mg/kg per day)	0.843	0.292
	hERG I inhibitor	No	No
	hERG II inhibitor	No	No
	Oral Rat Acute Toxicity LD50 (mol/kg)	2.024	2.518
	Oral Rat Chronic Toxicity LOAEL (log mg/kg bw per day)	2.318	2.049
	Hepatotoxicity	No	No
	Skin Sensitization	No	No
	*T pyriformis* toxicity (log µg/L)	0.34	0.285
	Minnow toxicity (log mM)	2.052	11.261

VDss, steady state volume of distribution; BBB, brain–blood barrier; CNS, central nervous system; OCT2, organic cation transporter 2; AMES, *Salmonella*/microsome mutagenicity assay; hERG, human ether-a-go-go gene; LOAEL, lowest dose of a compound that resulted in an observed adverse effect.

Regarding the medicinal chemistry tool and synthetic accessibility, monomethylsulochrin showed a suitable profile, which is very important in the molecular optimization process and for the design of a compound that can be obtained at affordable costs.

## Conclusion

This work showed that monomethylsulochrin has promising antileishmanial properties, as it can act against the promastigote form and is also active against the intracellular amastigote form. Furthermore, the study demonstrates, for the first time, the antileishmanial activity of monomethylsulochrin and suggests that the mitochondria of the parasites may be the main target organelle. In this article, docking analyses showed a possible mechanism of inhibition promoted by monomethylsulochrin in relation to the CYP51 enzyme. This enzyme is essential for the growth of *Leishmania*, as it acts in the oxidative removal of a methyl group from the 14-a position of lanosterol and plays an important role in preserving the integrity of the parasite membrane. Moreover, monomethylsulochrin would have favorable pharmacokinetics on application and possess drug-like properties. These results open new perspectives for the research of compounds isolated from endophytic fungi as well as to contribute to the development of new drugs for the treatment of leishmaniasis.

## Data availability statement

The original contributions presented in the study are included in the article/supplementary material. Further inquiries can be directed to the corresponding authors.

## Ethics statement

All the experiments were carried out in compliance with the National Council for Control of Animal Experimentation (Conselho Nacional de Controle de Experimentação Animal - CONCEA) and approved by the local Animal Use Ethics Committee (Comissão de Ética no Uso de Animais CEUA-IOC) license number L53/2016.

## Author contributions

Conceptualization, JVS-S, ASdO, and AMdRM; methodology, JVS-S, FA-S, AMdRM and ASdO; validation, KSC, FA-S, PSBM, AMdRM and ASdO; formal analysis, KSC, FA-S, ASdO, PSBM and AMdRM; investigation, JVS-S, RFM, LAW, CdSFdS, DJH, KFT, ARC, THD, JWdCJr; data curation, FA-S, NNT, ALB, PSBM, AMdRM and ASdO; writing—original draft preparation, JVS-S, ASdO, and AMdRM; writing—review and editing, all authors; visualization, JVS-S; supervision, KSC, FA-S and AMdRM; funding acquisition, KSC, FA-S and AMdRM. All authors have read and agreed to the published version of the manuscript.

## Funding

This research was funded by the Oswaldo Cruz Institute, by the Coordination for the Improvement of Higher Education Personnel (Coordenação de Aperfeiçoamento de Pessoal de Nível Superior do Brazil; CAPES), grant number 88887.368507/2019-00, Finance Code 001, and by the Carlos Chagas Filho Foundation for Research Support of the State of Rio de Janeiro (Fundação Carlos Chagas Filho de Amparo à Pesquisa do Estado do Rio de Janeiro; FAPERJ), grant number E-26/210.344/2019, E-26/201.765/2019 and E-26/211.680/2021 (269680). The CytoFlex flow cytometer used at Flow Cytometry Core Facility was acquired by FAPERJ, grant number E-26/110332/2014. The APC (Fund for Conjoint Research Project) was funded by the Oswaldo Cruz Institute/FIOCRUZ. FA-S is a postdoctoral research fellow and scholarship holder of CAPES, grant number 88887.363006/2019-00. KC is (CNPq 315225/2021-1) is senior researcher.

## Acknowledgments

We are particularly grateful to Thaize Quiroga Chometon and Vanessa de Abreu Costa from the Flow Cytometry Core Facility of the Oswaldo Cruz Institute, FIOCRUZ, Brazil for flow cytometry analyses.

## Conflict of interest

The authors declare that the research was conducted in the absence of any commercial or financial relationships that could be construed as a potential conflict of interest.

## Publisher’s note

All claims expressed in this article are solely those of the authors and do not necessarily represent those of their affiliated organizations, or those of the publisher, the editors and the reviewers. Any product that may be evaluated in this article, or claim that may be made by its manufacturer, is not guaranteed or endorsed by the publisher.
